# 

**Published:** 1910-10-29

**Authors:** 


					Just Issued. Ninth Edition, Rewritten and Enlarged, 9s. net.
PHARMACY, MATERIA MEDICA & THERAPEUTICS.
Being a Commentary on the B.P. and its Drugs, with their Preparations,
and on all the new Non-official Remedies in use, with Chapters on the
different processes used in Dispensing, Prescription-writing, Latin
Glossary, &c. By Sir W. WHITLA, M.D., LL.D., Professor, Materia
Medica, Queen's University, Belfast, and Senior Physician, Royal Victoria
Hospital.
By the same Author. New Work. 1,900 pages. Crown 8vo. 25s. net.
PRACTICE OF MEDICINE.
Containing in two Handy Volumes a complete and independent Article on
the Etiology, Symptoms, and Diagnosis of every Medical Disease; arranged
in Dictionary Form for Practitioners and Students.
London: BailliJire, Tindall & Cos, 8 Henrietta Street, W.O.
A MANUAL OF
MEDICAL EXERCISES.
By PERCY LEWIS, M.D., M.R.C.S.,
Hon. Medical Officer to Victoria Hospital, Folkestone.
66 pages, with 35 Illustrations, cloth, 1s. 6d. net; post free, Is. 7d.
H. K. LEWIS, 136 GOWER STREET, LONDON, W.C.
Appointments and Resignations.
Mr. Richard Parry has been appointed assistant house
surgeon to the Royal Albert Hospital, Devonport.
The managers of the Western Infirmary, Glasgow,
have made the following appointments : Dr. R. Speirs
Fullarton, assistant physician; Dr. Alexander MacLennan.
assistant surgeon; Dr. George Allison Allan and Dr.
James R. C. Greenlees, dispensary physicians; Dr. Robert
Carslaw, dispensary surgeon; Dr. W. A. Campbell and
Dr. A. J. Hutton, extra-dispensary surgeons.
At the last meeting of the Board ofr Management of the
Manchester Royal Infirmary the following appointments
were confirmed : Mr. Howard Buck, M.B., Ch.B. Vict.,
F.R.C.S. Eng., appointed assistant surgical officer; Mr.
A. J. Brown, M.R.C.S., L.R.C.P., appointed accident-room
house surgeon; Dr. A. Ramsbottom, re-appointed assistant
medical officer; Mr. H. H. Rayner, F.R.C.S. Eng., ap-
pointed assistant surgical officer; Mr. W. H. Hey, F.R.C.S.
Eng., re-appointed resident surgical officer; Mr. Cecil
Hibbert, M.B., Ch.B. Vict., appointed medical officer at
the Central Branch; Mr. J. W. Bride, M.B., Ch.B. Vict.,
M.B., B.S. Lond., medical officer at the Central Branch;
Mr. R. Ollerenshaw, F.R.C.S. Eng., appointed surgical
registrar; and Mr. W. R. Douglas, F.R.C.S. Eng., re-
appointed surgical tutor.
Obituary.
The death is announced, as the result of an accident,
of Mr. Richard Thompson Caesar, of Beareted, Kent, at
the age of seventy-two. Mr. Ctesar took his M.R. C.S. in
1859, and became an L.S.A. three years later.
14G THE HOSPITAL October 29, 1910.
Gifts and Bequests.
The Honorary Secretaries of King Edward's Hospital
.Fund for London have received at the Bank of England the
.sum of ?10,000 from an anonymous contributor.
Ms. Thomas Conway, of Wimbledon, has left ?100 to tbe
Royal Hospital for Incurables, Putney, S.W.
An anonymous donor has sent ?100 to the Central
.London Ophthalmic Hospital, Gray's Inn Road.
Mr. William Alfred Pryor, of Chesham, Bucks, has
bequeathed ?100 to the Willesden Cottage Hospital.
Mrs. Catherine Agnes Carr, of Maldon Road, Preston,
-Sussex, has left ?500 to the Brighton and Hove Dispensary.
The treasurers of the Middlesex Hospital Cancer Charity
ihave received ?200, the proceeds of the students' annual
.smoking concert.
The Treasurers of the Middlesex Hospital Cancer Charity
have received ?1,000 from Mrs. Clara. Cumines to name a
'bed in perpetuity.
Tee Treasurer of the Leicester Infirmary has received
?184 10s. 4d. from the employees of the British United
Shoe Machinery Co.
Mrs. Mary Jane Whitney, of Monmouth, has left ?100
?each to the Hereford General Hospital and the Hereford
Eye and Ear Hospital.
Mr. Eli Townend, of Calder Villa, Ossett, Yorks, retired
mungo manufacturer, has left ?20 per annum for ten years
to the Ossett Nursing Association.
The Prince of Wales has sent an annual subscription
?of ?25 to the Seamen's Hospital Society (Dreadnought
Hospital, Greenwich) for the sick and disabled sailors of
the Empire.
The Rev. John Barrett, of Cork, Roman Catholic priest,
who has left personal estate valued at ?12,549, has be-
queathed ?20 to the North Charitable Infirmary, Cork,
:and ?1 Is. per annum for five hundred years after his death.
Me. William Hall, of Highbury Quadrant, has be-
queathed ?500 to the Lincoln County Hospital, and ?1,4C0
"to his trustees for distribution among the Great Northern
?Central Hospital, the Home for Confirmed Invalids,
Aubert Park, the Royal Hospital for Incurables, Putney,
and seven other charities.
The family of the late Thomas Creaser Kellock, Esq.,
ha? given the sum of ?500 to the endowment fund of
'the Totnes Cottage Hospital in memory of their father;
-a like amount (?500) was given by Mr. Kellock's brother,
Dr. W. B. Kellock, F.R.C.S., in 1901, and ?500 left by
him in his will was paid by the residuary legatees in 1903.
Among the latest contributions to King Edward's Hospital
Fund for London are : Annual subscriptions, Mr. Francis
Reckitt, ?52 10s.; "W.," ?50; Rev. A. W. Davies,
.?25; Mrs. Hudson, ?25: Messrs. Macmillan and Co.,
Limited, ?25; Goldsmiths' and Silversmiths' Company,
Limited, ?21. Donations, Mr. C. W. Drabble, ?105;
Lady Camilla Fortescue, ?20.
Cai>tain Henry Boyles Murray, of Bina Gardens, South
Kensington, who left estate valued for probate at
?125,921 7s. 9d. gross, has bequeathed ?1,050 to the West
London Hospital for the endowment of a bed, ?1,000 each
-to St. Thomas's Hospital and St. George's Hospital for a
like purpose, ?1,000 each to King's College Hospital and
St. Mary's Hospital, ?500 each to the Hospital for Children,
'Great- Ormond Street, and the Victoria Hospital for
-Children, Queen's Road, Chelsea, for the endowment of
.cots, and ?500 each to the Royal Dental Hospital and
-the London Fever Hospital, Liverpool Road, N., for general
ipurposes, and ?100 to the Cancer Hospital, Fulham Road,
for general purposes.
Jottings.
The Queen has sent a present of cast linen for the use
of the patients in St. George's Hospital.
The exhibition of pictures in aid of the funds of the
Middlesex Hospital at the Victoria Gallery, Victoria Street,
S.W., closes to-day (Saturday).
The Middlesex Hospital Students' annual smoking con-
cert in aid of the cancer charity, which was fixed for
November 18 at the Queen's Hall, will not take place this
year.
It has been decided at a county meeting held at Maid-
stone to enlarge and endow the West Kent General Hos-
pital as a memorial to King Edward. It is hoped to raise
?20,000. Towards this sum Mr. H. G. Kleinworth has
promised ?2,000 and Sir Marcus Samuel ?1,000.
Vice-presidents of the League of Mercy are asked to
send their collections to the presidents and lady presidents
by November 15, and presidents and lady presidents to the
head office by December 15, so that the full grant to the
King Edward's Hospital Fund may be given by the end
of the year.
Sir Charles Wakefield will open the sale of patients'
work at the British Home and Hospital for Incurables,
Streatham, on Wednesday, November 23, at 3 o'clock.
The money raised at this sale is distributed among the
patients, and thus provides them with a little pocket-
money for the year.
It has been resolved that of the funds collected for a
Surrey memorial, ?500 should be devoted to the erection of
a statue of the late King, and that ?5,000 should be spent
on the endowment of the Children's Convalescent Home for
Surrey, the Home to be known hereafter as the King
Edward VII. Convalescent Home for Surrey Children.
A large meeting has been held in the Shire Hall, Wor-
cester, presided over by the Lord-Lieutenant (Lord
Coventry), to consider whether the proposed county
memorial to the late King should take the form of either
a fund to benefit the Worcester General Infirmary and
Kidderminster Hospital, or an extension of the sanatorium
for the treatment of consumption at Knightwick. This
latter proposal was decided upon after some discussion.
A few weeks ago the late Prince Francis of Teck con-
sented to become Vice-President of the Homes for Little
Boys. The Prince was especially anxious that a little boy
named Arthur William Wilson, aged seven, might bo
successful at the December election. The boy's father
died of consumption last January after many years of
suffering, and his mother is in a lunatic asylum. The
committee proposes to admit the child into one of the
homes at once.
The Bishop of Oxford the other day, in presenting to
students in the London School of Dental Surgery the
prizes competed for in the session 1909-10, acknowledged
the good work of the Royal Dental Hospital of London,
and said that as the profession of surgery had advanced in
dignity and distinction so would dental surgery advance.
It had advanced greatly since the days when a man com-
bined the diverse occupations of drawing teeth, letting
blood, and mending tea-kettles.
TO HOSPITAL SECRETARIES AND OTHERS.
We invite contributions from any of our readers, and
Bhall be glad to pay, according to length, for items of news
for the institutional section (including appointments, resig-
nations, gifts, etc.) which we may publish. All payments
for manuscripts used are made as early as possible after the
beginning of each quarter.
LEAGUE OF MERCY MEETINGS FOR THE WEEK.
Thursday, November 3, Strand District : Special
matinee of " The Dollar Princess," Daly's Theatre, 2.15
P.M.
Thursday, November 3, South St. Pancras District :
Mrs. George Alexander "At Home," 57 Pont Street, 4 to
6 P.M.
Saturday, November 5, Westminster District : Enter-
tainment at Napier Hall, 8 p.m.
INHERITANCE OF MENTAL CHARACTERS.
Dr. H. B. Donkin delivered the Harveian Oration at the
Royal College of Physicians last week, choosing as his
subject " Inheritance of Mental Characters." Sir Thomas
Barlow presided. In the course of his oration, Dr. Donkin
protested against the Mendelian hypothesis of " unit segre-
gation " being termed a " discovery" and employed as a
fact in argument. The terms "criminology," "mental
defect," and " eugenics " illustrated the necessity of clear
conceptions when speaking of the difference between
"innate" and "acquired" "characters." The only im-
portant link between the study of crime and that of heredity
was the considerably larger minority of persons with clearly
mental defects, apparently congenital, found among con-
victed criminals than in the population at large. There was
much loose talk about so-called " degeneracy," the only
legitimate meaning of which denoted " racial " change. No
really rational hypothesis of hereditary criminality had been
even formulated. To a large extent the two brain
mechanisms, the "instinctive" and the "individually
acquired," were in opposition. Capacity for making mental
acquirements was innate and transmissible, but for its de-
velopment it required appropriate stimulus. Man's mind,
like his body, was certainly both born and made, but his
adult mind was much more made than born. The manifest
bearing of this truth was to emphasise the paramount im-
portance of education.
THE MEDICAL LIBRARY.
Post Free.
" Medical History: From the Earliest Times." By
E. T. Withington, M.A., M.B. Oxon ... ... 12s. 10cl.
" The Consumptive Working Man. What Can the
Sanatoria Do for Him? " By Noel D. Bards-
well, M.D.  Is. 9d.
" Photomicrography." By Edmund Spitta,
M.R.C.S. With 41 Half-tone Reproductions
from original negatives and other Illustrations 12s. Od.
Of all booksellers or of The Scientific Press, Limited*
28 and 29 Southampton Street, Strand, London, W.C.
THE HOSPITAL October 29, 1910.
Name
Address
This Coupon must accompany manuscript or contributions in-
tended for The Hospital.
BUSINESS NOTICES.
Annual Subscriptions.
The rate of annual subscriptions to The
Hospital, either direct from the Office or through
agents, is: ?
For the United Kingdom  15s. a year.
For the Colonies and Abroad ... 17s. ,,
For the United Kingdom (with
Insurance Policy)  ?1 ,,
inclusive of postage in each case.
Letters relating to the Publishing, Sale and
Advertisement Departments must be addressed to
the Manager (not to the Editor): ?
The Manager,
The Hospital Building,
28 and 29 Southampton Street,
Strand, London, W.C.
Books for Review.
Publishers are particularly requested to send
advance proofs of any new books of importance,
whenever possible, as the Editor has. made arrange-
ments to publish immediate reviews, and on a new
plan.
THE IDEAL WORK OF REFERENCE.
BURDETT'S HOSPITALS & CHARITIES
1910 EDITION.
Zhe JDeaivJSook of philanthropy anb Ibospltal annual.
Edited by SIR HENRY BURDETT, K.C.B., K.C.V.O.
A Complete Guide and Directory of the Hospitals, Asylums, and Charitable Institutions
throughout the English-speaking World.
Contains Special New Chapters on :?
Voluntary Hospitals in 1909.?Soundness of the Voluntary System.?The need of greater zeal on the
part of those who accept office.?State-aided Hospitals in the U.S.A. and Canada.?The Appropriation
of Public Funds for the partia^Support of Voluntary Hospitals in U.S.A. and Canada.?The Need of an
Apostle and of greater interest on the part of the Clergy, &c., &c.
TWENTY-FIRST YEAH OF PUBLICATION.
Over 1,000 pages. Price 10/6 net; Post Free, 10/11.
ORDER your copy of the Medical Whitaker NOW.
Publishers :
THE SCIENTIFIC PRESS, LIMITED, 28 & 29 Southampton Street, Strand, London, W.C.

				

